# *Arabidopsis thaliana* Contains Both Ni^2+^ and Zn^2+^ Dependent Glyoxalase I Enzymes and Ectopic Expression of the Latter Contributes More towards Abiotic Stress Tolerance in *E*. *coli*

**DOI:** 10.1371/journal.pone.0159348

**Published:** 2016-07-14

**Authors:** Muskan Jain, Rituraj Batth, Sumita Kumari, Ananda Mustafiz

**Affiliations:** 1 Faculty of Life Sciences and Biotechnology, Plant Molecular Biology Laboratory, South Asian University, Akbar Bhawan, Chanakyapuri, New Delhi 110021, India; 2 Sher-e-Kashmir University of Agricultural Sciences and Technology, Jammu 180009, India; CSIR-National Botanical Research Institute, INDIA

## Abstract

The glyoxalase pathway is ubiquitously found in all the organisms ranging from prokaryotes to eukaryotes. It acts as a major pathway for detoxification of methylglyoxal (MG), which deleteriously affects the biological system in stress conditions. The first important enzyme of this system is Glyoxalase I (GLYI). It is a metalloenzyme which requires divalent metal ions for its activity. This divalent metal ion can be either Zn^2+^ as found in most of eukaryotes or Ni^2+^ as seen in prokaryotes. In the present study, we have found three active GLYI enzymes (AtGLYI2, AtGLYI3 and AtGLYI6) belonging to different metal activation classes coexisting in *Arabidopsis thaliana*. These enzymes have been found to efficiently complement the *GLYI* yeast mutants. These three enzymes have been characterized in terms of their activity, metal dependency, kinetic parameters and their role in conferring tolerance to multiple abiotic stresses in *E*. *coli* and yeast. AtGLYI2 was found to be Zn^2+^ dependent whereas AtGLYI3 and AtGLYI6 were Ni^2+^ dependent. Enzyme activity of Zn^2+^ dependent enzyme, AtGLYI2, was observed to be exceptionally high (~250–670 fold) as compared to Ni^2+^ dependent enzymes, AtGLYI3 and AtGLYI6. The activity of these GLYI enzymes correlated well to their role in stress tolerance. Heterologous expression of these enzymes in *E*. *coli* led to better tolerance against various stress conditions. This is the first report of a higher eukaryotic species having multiple active GLYI enzymes belonging to different metal activation classes.

## Introduction

Methylglyoxal (MG) is produced during normal metabolism of glucose and lipid but has been reported to increase in stress conditions in animals, mammals, yeast and bacterial systems [1,2]. MG levels have been found to increase by 2–6 folds in response to the abiotic stresses in plant system [3]. Higher concentration of MG is detrimental to the system, as it is a potent cytotoxin and reacts with major macromolecules including DNA, RNA [4], proteins [5] and also inhibits cell proliferation [6]. Glyoxalase system is majorly involved in detoxification of MG. Glyoxalase pathway consists of two enzymes, Glyoxalase I (GLYI) and Glyoxalase II (GLYII). GLYI uses GSH as a cofactor for the conversion of MG to S-D-lactoylglutathione (SLG) and GLYII acts upon SLG and leads to the production of D-lactate, giving GSH back to the system [7,8]. The glyoxalase system is ubiquitous and is found throughout biological life [9]. The widespread distribution and presence of glyoxalase system in living organisms suggests that it might be fulfilling a function of fundamental importance to biological life [7]. In many studies it has been shown that glyoxalase pathway plays important role in stress tolerance. Overexpression of *GLYI* and *GLYII* genes was shown to confer abiotic stress tolerance in tobacco [10]. Therefore, a detailed investigation of the glyoxalase pathway in plants is required so as to generate stress tolerant varieties of plants. GLYI, the first enzyme of glyoxalase pathway, is broadly categorized into Zn^2+^ dependent and non-Zn^2+^ dependent class of metal activation. The Zn^2+^ dependent GLYI enzymes are thought to be of eukaryotic origin as studied in *Homo sapiens* and *Saccharomyces cerevisiae* [11,12] and non-Zn^2+^ dependent GLYI enzymes are thought to be of prokaryotic origin as seen in *Pseudomonas aeruginosa*, *Neisseria meningitidis* and *Yersinia pestis* [13]. The coexistence of both the classes of enzyme in *Pseudomonas aeruginosa* [14] and the characterization of a Ni^2+^ dependent GLYI from rice (a eukaryote) [15] has led to the discouragement of the view that Zn^2+^ dependent GLYI belongs to eukaryotes and non-Zn^2+^ dependent GLYI exists only in prokaryotes.

In the genome wide analysis, eleven *GLYI* genes were found to exist in *Arabidopsis thaliana* [16]. These GLYI family members have been found to be differentially regulated in response to various stress conditions and developmental stages. However, further *in silico* analysis revealed that out of eleven GLYI, only three were active and contained all domains required for glyoxalase activity, i.e. AtGLYI2, AtGLYI3 and AtGLYI6 (S1 Table). Out of three, AtGLYI2 has been predicted to be Zn^2+^ dependent and AtGLYI3 and AtGLYI6 have been predicted to be non-Zn^2+^ dependent. Arabidopsis is unique due to the presence of multiple active GLYI with different metal selectivity as no other eukaryotic organism has been reported to have GLYI with both metal activation classes till now. In the present study, we have characterized all the three active AtGLYI enzymes in terms of their metal dependency and kinetic parameters. On heterologous expression, they have been found to provide stress tolerance in *E*. *coli*, thus, indicating their role in stress tolerance. They also successfully complemented the yeast *GLYI* mutant cells and provided them tolerance against high concentrations of MG.

## Materials and Methods

### *In-silico* characterization of *GLYI* gene family in *Arabidopsis thaliana*

The bioinformatics analysis in *Arabidopsis thaliana* revealed the presence of eleven *GLYI* genes [16]. To find out active GLYI members, each of the 11 AtGLYI protein sequences was analyzed using BLASTP (http://blast.ncbi.nlm.nih.gov/Blast.cgi). The metal dependency of the putatively active AtGLYI proteins was analyzed by aligning their protein sequences with those of other known GLYI protein from different species on CLUSTALW (http://www.genome.jp/tools/clustalw). The multiple sequence alignment was visualized using Jalview [17].

### Cloning of all putatively active *AtGLYI* genes

Total RNA was isolated from fresh Arabidopsis leaf tissue using IRIS Kit (Bangalore, Genei) according to manufacturer’s instructions. The RNA was reverse transcribed using the RevertAid H Minus first stand cDNA synthesis Kit (Fermentas Life Sciences, USA). This first strand cDNA was used to amplify *AtGLYI2*, *AtGLYI3* and *AtGLYI6* genes using respective gene specific primers having EcoRI site in forward primers and XhoI site in reverse primers using Q5 DNA polymerase (NEB). The sequences of these primers are listed in S2 Table. The PCR product of all three *GLYI* genes carrying EcoRI and XhoI sites were cloned into blunt end pJET cloning vector (CloneJET^™^PCR cloning Kit, Fermentas Life Sciences, USA). Once the correct sequence of all *GLYI* genes was confirmed, they were subcloned into pET28a expression vector to express the recombinant proteins with N-terminal Histidine tag.

### Expression and purification of active GLYI proteins from *Arabidopsis thaliana*

BL21 strain of *E*. *coli* containing the recombinant construct of pET28a and *GLYI* genes was used to express the proteins. For AtGLYI2, BL21+(pET28a-*AtGLYI2*) cells were grown till the optical density of 0.5 and then, induced with 0.5mM IPTG and grown at 37°C for 4 h. BL21+(pET28a-*AtGLYI3*) and BL21+(pET28a-*AtGLYI6*) cells were grown till an optical density of 0.5 and then induced with 0.05 mM IPTG for 15 h at 18°C. The cells were harvested and the expressed recombinant proteins with N-terminal His tag were purified using Ni-NTA affinity chromatography. The purification profile of all the GLYI proteins was confirmed by running purified proteins on denaturing SDS gel.

### GLYI activity and metal dependency

In order to check the activity of AtGLYI3 and AtGLYI6, a standard enzyme assay mixture was prepared with 20 mM phosphate buffer of pH 8.0 containing 3.5 mM MG, 1.7 mM GSH and 20 μM NiCl2. The purified proteins were added to this mixture and a timescan was performed spectrophotometrically, taking the OD_240_ at every 10 seconds within a timescale of ~2 min. Since, AtGLYI3 and AtGLYI6 were predicted to be Ni^2+^ dependent enzymes, therefore the N- terminal Histidine tag having high affinity for nickel ions was removed using Thrombin Cleavage Kit (Sigma Aldrich, USA) and GLYI activity of all Histidine tag removed GLYI proteins was re-checked.

As Zn^2+^ dependent GLYI enzymes have very tightly bound metal ions, therefore the prebound metal ion from purified AtGLYI2 was first removed by dialyzing twice against EDTA and then dialyzing thrice against chelex treated MOPS. Once the metal ions were completely removed, the AtGLYI2 apoenzyme was reconstituted with different metal ions and GLYI activity was checked. For the preparation of chelex treated MOPS, 10 ml from 10% suspension of chelex was added in 1 L of MOPS buffer and stirred overnight. For reconstitution of AtGLYI2, 5 μM of the apoenzyme was incubated with 25 μM of various metal chlorides at 4°C. GLYI activity of reconstituted protein fractions was measured after 2 h and 4 h.

### Determination of kinetic parameters of AtGLYI enzymes

The purified proteins were firstly quantified using Bradford assay with BSA (Bovine serum albumin) as a standard. The pH profiling of AtGLYI proteins was done by measuring the activity of purified proteins over a wide range of pH in different buffers (pH 5.5–6.75: MES buffer, pH 6.5–8.0: MOPS buffer, pH 7.40–9.0: Tris buffer). The observed activity for all three AtGLYI enzymes was plotted against pH.

Kinetic parameters such as Michaelis-Menten constant (K_m_), catalytic efficiency (K_cat_) and specific activity were calculated by measuring the rate of formation of SLG. For AtGLYI3 and AtGLYI6, GLYI assay buffer was prepared with GSH concentration of 0.01–0.15 mM. All reactions were initiated by the addition of enzyme and rate of formation of SLG was measured spectrophotometrically at 240 nm after every 10 s for 2 min. The enzyme activity at different concentrations of GSH was measured in triplicates and the values were averaged to obtain final parameters. For AtGLYI2, the purified enzyme was diluted and assayed under similar conditions as AtGLYI3 and AtGLYI6 but with different GSH concentration ranging from 0.01–0.25 mM.

### Abiotic stress tolerance assay of *E*. *coli* cells overexpressing *AtGLYI* genes

BL21 cells containing expression constructs such as pET28a-*AtGLYI2*, pET28a-*AtGLYI3* and pET28a-*AtGLYI6* were grown in Luria Bertani medium at 37°C. BL21 with empty vector (pET28a) was used as a control. As the culture reached its mid exponential phase with optical density of 0.5, the cells were subjected to different stress conditions such as 200 mM NaCl, 5 mM H_2_O_2_, 0.5 mM MG, 1mM MG, 100 mM Mannitol and 42°C heat stress and induced with 0.5 mM IPTG and allowed to grow at 37°C. The growth pattern of control as well as stress treated cells was noted for 12 h by taking OD at 600 nm after every 2 h time point. The data obtained in triplicates was averaged and used to plot the graph.

### Functional complementation of yeast *GLYI* mutants

The full length *AtGLYI* genes were cloned in yeast expression vector pYES2 under the control of galactose inducible and glucose repressible GAL1 promoter. The *ΔGLO1* mutant BY4741 cells were transformed with expression construct of pYES2-*AtGLYI2*, pYES2-*AtGLYI3*, pYES2-*AtGLYI6* and empty vector pYES2. The positive transformants were picked via Ura- prototrophy. To check the expression of AtGLYI proteins, total yeast proteins were isolated followed by western blotting using the protein specific antibodies [18].

The transformed cells were streaked on Ura- solid synthetic defined media containing either 20% glucose or 20% galactose with different MG concentrations (0.0 mM-4.0 mM MG) and grown at 30°C for 78 h. For comparing the growth profile of these constructs, the transformed cells were grown in Ura- liquid SD media and growth was monitored by taking OD at 600 nm at 5 h interval for 30 h. The entire experiment was replicated and obtained data was averaged to plot the graph.

## Results

### Presence of multiple active GLYI proteins from different metal activation class in *Arabidopsis thaliana*

The genome wide analysis of GLYI family members in Arabidopsis genome revealed the presence of eleven *GLYI* genes [16]. By using BLASTP search tool, each of these eleven gene members were individually analyzed for the presence of various important domains required for the GLYI activity. These important domains include metal binding site, glutathione binding site, dimer interface and the active site for catalyzing the reaction. Out of eleven, only three *AtGLYI* genes such as *AtGLYI2*, *AtGLYI3* and *AtGLYI6* were found to have both the metal binding sites and active sites and were predicted to encode active proteins, whereas others lacked one or more of these domains (S1 Table). Thus, three putatively active GLYI proteins were identified in *A*. *thaliana*.

**Fig 1:**
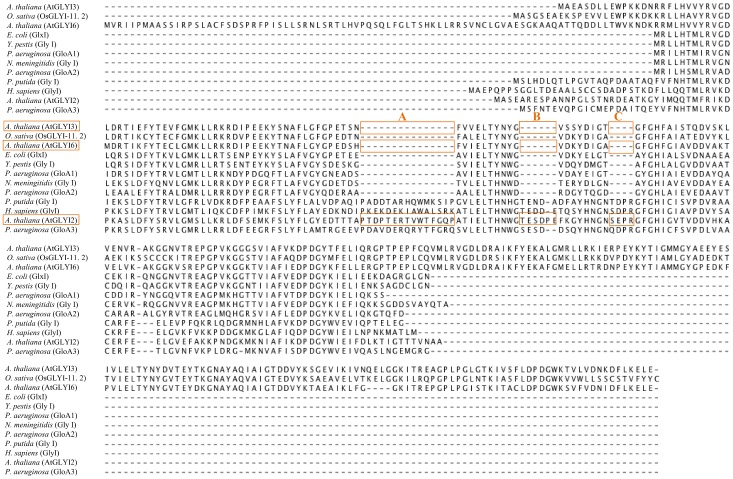
Sequence alignment of AtGLYI proteins with GLYI proteins from different species. Protein sequences of AtGLYI2, AtGLYI3 and AtGLYI6 (Acc. No. NP_172291.1, NP_172648.1, NP_176896.1 respectively) were aligned with GLYI protein sequences from *O*. *sativa* (Os08g09250), *E*. *coli* (AAC27133), *Y*. *pestis* (YP_002347347), *P*. *aeruginosa* (GloA1, GloA2 and GloA3 with Acc. No. AAG06912, AAG04099 and AAG08496 respectively), *N*. *meningitidis* (NP_273389.1), *H*. *sapiens* (NP_006699) and *P*. *putida* (NP_745896). The letters A, B and C represent extended sequences found in Zn^2+^ dependent GLYI and absent in non-Zn^2+^ dependent GLYI.

In order to predict the metal dependency of these three putatively active AtGLYI proteins, a sequence alignment of protein sequences of AtGLYI2, AtGLYI3 and AtGLYI6 with the known GLYI from *Oryza sativa*, *Escherichia coli*, *Yersinia pestis*, *Pseudomonas aeruginosa* (GloA1, GloA2, GloA3), *Pseudomonas putida*, *Neisseria meningitidis and Homo sapiens* was done using CLUSTALW tool (Fig 1). This revealed the presence of an extended amino acid sequence in AtGLYI2 which is characteristic of Zn^2+^ dependent GLYI whereas in case of AtGLYI3 and AtGLYI6, this extra amino acid sequence was missing as seen in other non-Zn^2+^ dependent GLYI proteins. Therefore, AtGLYI2 was predicted as Zn^2+^ dependent GLYI and AtGLYI3 and AtGLYI6 as non-Zn^2+^ dependent GLYI.

### AtGLYI2 is Zn^2+^ dependent; AtGLYI3 and AtGLYI6 are Ni^2+^ dependent GLYI enzymes

**Fig 2:**
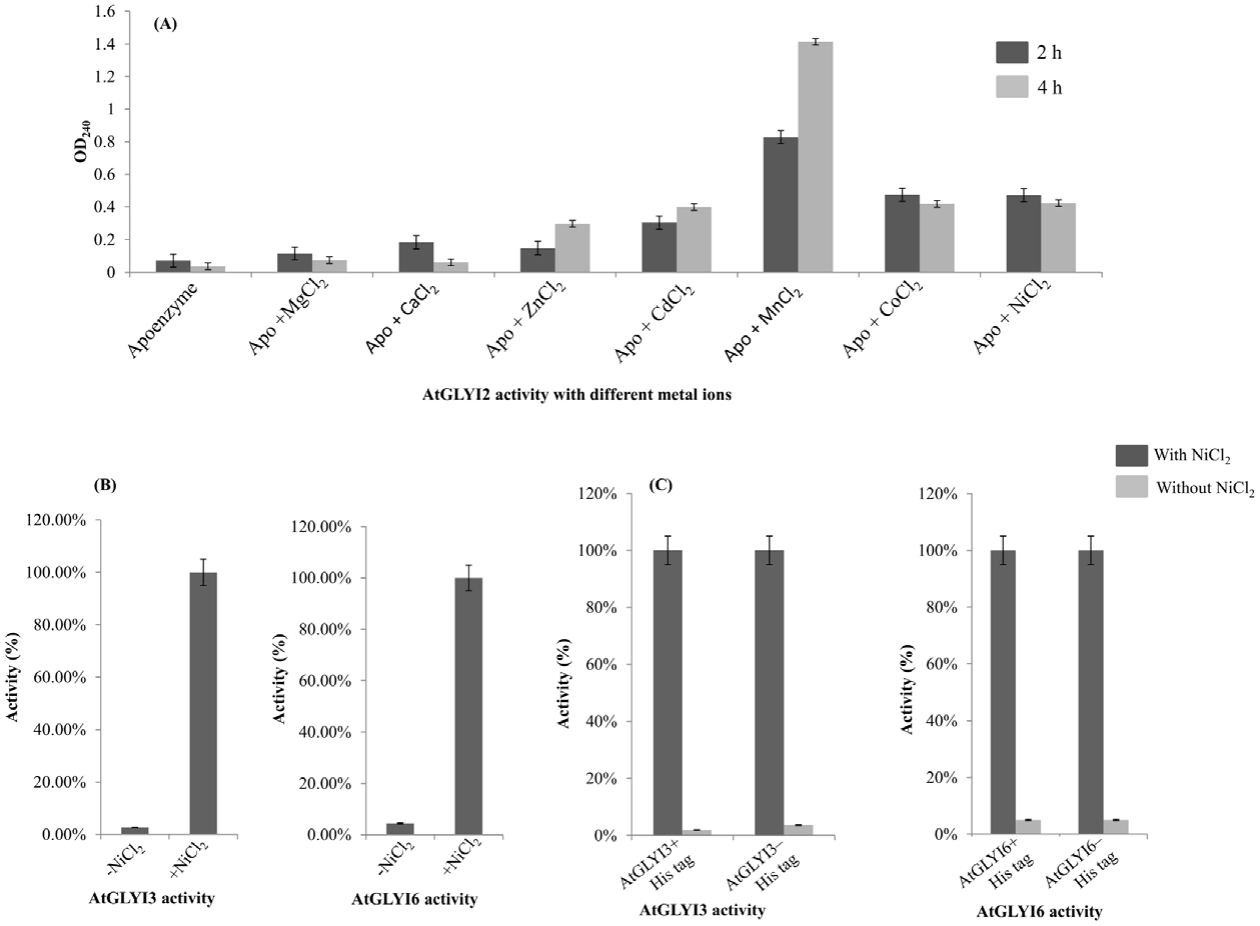
AtGLYI2 is Zn^2+^ dependent while AtGLYI3 and AtGLYI6 are Ni^2+^ dependent GLYI. (A) Histogram depicting activity of purified AtGLYI2 protein in presence of various metal ions. AtGLYI2 was demetalled using EDTA and then, reconstituted with different metal ions. The activity was checked after 2 h and 4 h of reconstitution. AtGLYI2 showed maximal activity with MnCl_2_ followed by CoCl_2_, NiCl_2_ and CdCl_2_. This behavior is characteristic of Zn^2+^ dependent GLYI enzymes. (B) Histogram depicting activity of purified AtGLYI3 and AtGLYI6 in presence and absence of Ni^2+^. (C) Histogram depicting activity of thrombin cleaved and purified AtGLYI3 and AtGLYI6 (to remove His tag) in presence and absence of Ni^2+^.

AtGLYI2 has been predicted as Zn^2+^ dependent GLYI. AtGLYI2 apoenzyme was reconstituted with different metal ions and activity was checked. AtGLYI2 behaves like known Zn^2+^ dependent GLYI from other organisms showing maximal activity with MnCl_2_ followed by CoCl_2_, NiCl_2_ and CdCl_2_ (Fig 2A). Thus, AtGLYI2 can be considered as a Zn^2+^ dependent GLYI. AtGLYI3 and AtGLYI6 have been predicted as Ni^2+^ dependent enzymes. GLYI assay for AtGLYI3 and AtGLYI6 was performed with purified proteins in the absence and presence of Ni^2+^ ion. In presence of Ni^2+^, activity was found to be increased substantially (100 fold) (Fig 2B).

Thus, both AtGLYI3 and AtGLYI6 can be considered as Ni^2+^ dependent enzymes. Since the recombinant AtGLYI enzymes were purified using a His tag, there is a possibility of a false activity of both the AtGLYI enzymes due to the affinity of Ni^2+^ ion for Histidine tag. To rule out this probability, the N-terminal Histidine tag of AtGLYI3 and AtGLYI6 proteins was removed. The GLYI activity of proteins without N-terminal Histidine was observed to be similar to the activity of AtGLYI proteins with N-terminal Histidine tag (Fig 2C). Therefore, this result suggests that there was no contribution of Histidine tag in the GLYI activity of AtGLYI3 and AtGLYI6.

### pH optima and enzyme kinetics of AtGLYI2, AtGLYI3 and AtGLYI6 enzymes

**Fig 3:**
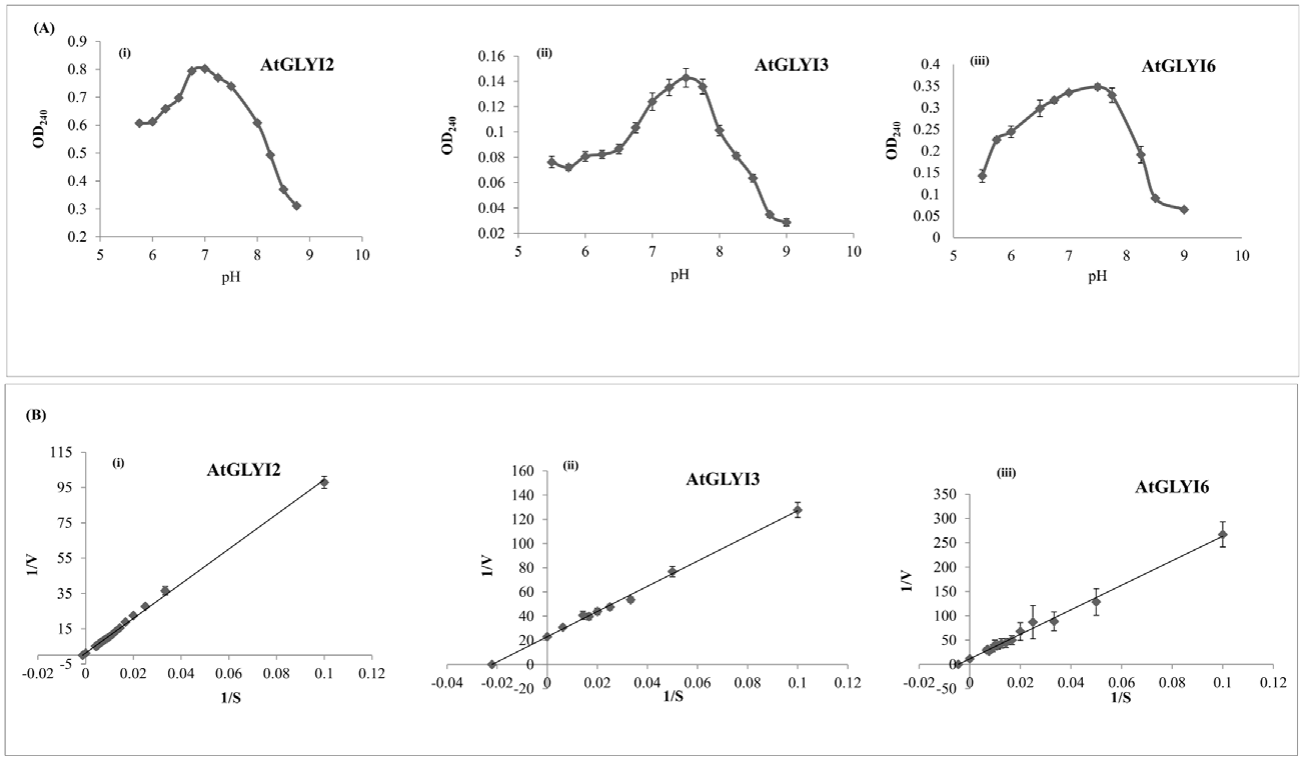
Kinetic parameters of purified AtGLYI proteins. (A) Upper panel shows graphs depicting effect of change in pH on the activity of (i) AtGLYI2, (ii) AtGLYI3 and (iii) AtGLYI6 proteins. A range of pH conditions analyzed and the corresponding absorbance indicative of protein activity is shown on X-axis and Y-axis, respectively. All the three proteins were purified using Ni-NTA affinity chromatography. The activity of purified proteins was checked over a wide range of pH in different buffers (pH 5.5–6.75: MES buffer, pH 6.5–8.0: MOPS buffer, pH 7.40–9.0: tris buffer). The optimal pH for AtGLYI2 is 7.0; AtGLYI3 is 7.5 and AtGLYI6 is 7.5. (B) Lower panel shows graphs depicting kinetic parameters for (i) AtGLYI2, (ii) AtGLYI3 and (iii) AtGLYI6. These parameters were calculated using Lineweaver Burk’s plot. GLYI assay was performed with purified proteins with varying concentration of GSH and increase in absorbance (240 nm) with time was monitored. From the data obtained, Lineweaver Burk’s plot was made and various kinetic parameters were calculated.

The activity of purified AtGLYI2, AtGLYI3 and AtGLYI6 proteins was checked in different buffers with a wide range of pH. The pH optima of AtGLYI2, AtGLYI3 and AtGLYI6 was found to be 7.0, 7.5 and 7.5, respectively (Fig 3A). The kinetic profile of the three AtGLYI enzymes was measured as described in Materials and Methods and Lineweaver Burk’s plots were made from the obtained data which was used to calculate the kinetic parameters such as K_m_, K_cat_, K_cat_/K_m_ and specific activity (Fig 3B). The K_m_ value of the enzymes AtGLYI2, AtGLYI3 and AtGLYI6 was found to be 786.78 μM, 45.320 μM and 223.015 μM, respectively. The specific activity of AtGLYI2, AtGLYI3 and AtGLYI6 was found to be 5157 μmol/min/mg, 20.7 μmol/min/mg and 7.68 μmol/min/mg, respectively. The values of all the calculated parameters are shown in Table 1.

**Table 1:** Kinetic parameters calculated for AtGLYI enzymes of *Arabidopsis thaliana*.

Kinetic Parameters	AtGLYI2	AtGLYI3	AtGLYI6
**Specific activity (μmol/min/mg)**	5157	20.7	7.68
**K**_**m**_ **(μM)**	786.78	45.320	223.015
**K**_**cat**_ **(s**^**-1**^**)**	1376X10^2^	7.28X10^2^	3.30X10^2^
**K**_**cat**_**/K**_**m**_ **(M**^**-1**^**/s**^**-1**^**)**	174.9X10^6^	16.08X10^6^	1.48X10^6^

### Heterologous expression of *AtGLYI* genes confer abiotic stress tolerance in *E*. *coli*

**Fig 4:**
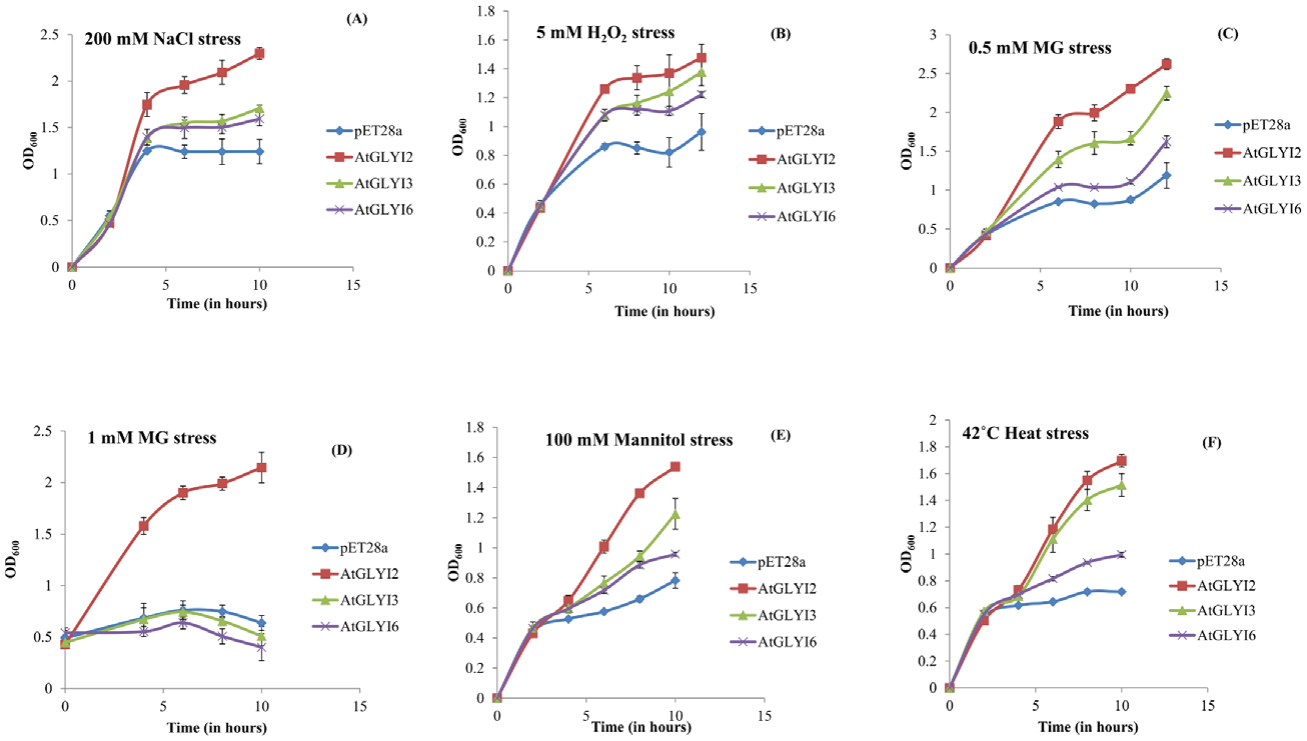
Stress tolerance assay of *E*. *coli* expressing *AtGLYI* genes. The *E*. *coli* cells transformed with *AtGLYI2* (shown in red color), *AtGLYI3* (shown in green color) and *AtGLYI6* (shown in purple color) genes were grown in presence of (A) salinity stress (200 mM NaCl), (B) oxidative stress (5 mM H_2_O_2_), (C) Cytotoxic methylglyoxal stress (0.5 mM), (D) Cytotoxic methylglyoxal stress (1 mM), (E) Osmotic stress (100 mM mannitol), (F) Heat stress (42°C) and the growth was monitored over time. Cells containing empty vector construct (pET28a without any gene) were used as control (shown in blue color).

The BL21 strain of *E*. *coli* cells containing *GLYI* genes (*AtGLYI2*, *AtGLYI3* and *AtGLYI6*) cloned in pET28a expression vector were grown in presence of different stress conditions, such as 200 mM NaCl (salinity stress), 5 mM H_2_O_2_ (oxidative stress), 0.5 mM MG (exogenous methylglyoxal), 1 mM MG (exogenous methylglyoxal), 100 mM Mannitol (osmotic stress) and 42°C (heat stress). The growth pattern suggested that the cells over expressing *AtGLYI* genes grew better as compared to the cells with empty pET28a vector control (Fig 4). Among the three *GLYI* genes, *AtGLYI2* overexpressing cells were most tolerant to all the stress conditions followed by *AtGLYI3* and *AtGLYI6* respectively. In presence of 1 mM exogenous MG, only *AtGLYI2* overexpressing cells were able to survive; all other cells could not grow at all and remained in the stationary phase. In response to other stresses, *AtGLYI2* showed significant advantages over others as the cells overexpressing it grew 1.75–5 folds better than empty vector containing cells. Thus, *AtGLYI* genes have been found to provide tolerance to multiple abiotic stresses in *E*. *coli*.

### Functional complementation of yeast *GLYI* mutant (*ΔGLO1*) with *AtGLYI2*, *AtGLYI3* and *AtGLYI6*

**Fig 5:**
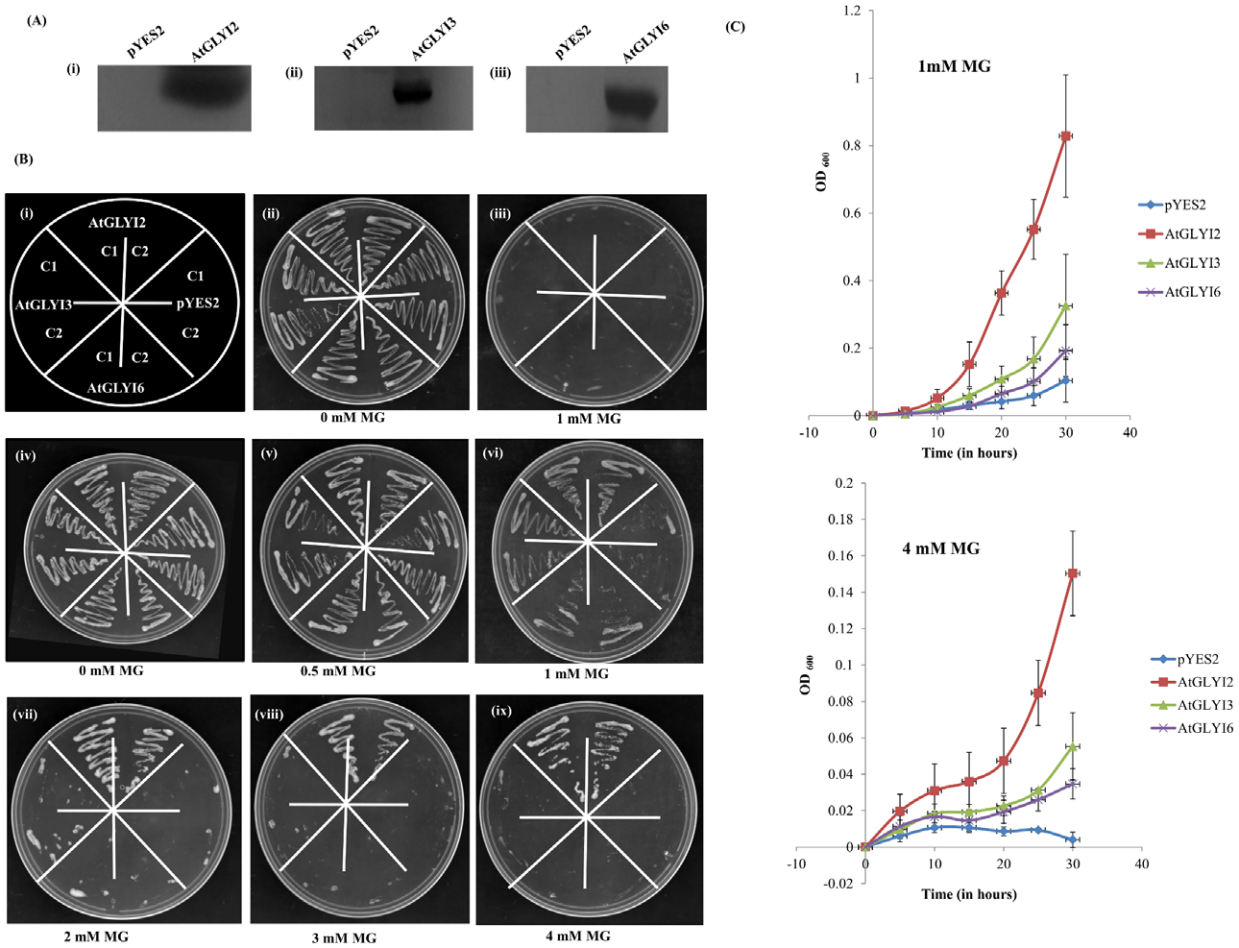
Functional complementation assay using the yeast *ΔGLO1* mutant. The *Saccharomyces cerevisiae GLYI* mutant cells were transformed with constructs pYES2-*AtGLYI2*, pYES2-*AtGLYI3*, pYES2-*AtGLYI6* and empty vector pYES2. (A) Western blot depicting expression of AtGLYI2, AtGLYI3 and AtGLYI6 proteins in the total protein extract of yeast cells. Western blotting was done using protein specific antibodies. (B) The transformed cells were grown on solid Ura- SD media with various supplements to check for stress tolerance of yeast cells transformed with *AtGLYI* genes (i) Pictorial depiction of various strains used (C1 and C2 refers to the two different colonies used). (ii) Medium supplemented with 20% glucose; (iii) Medium supplemented with 20% glucose and 1 mM MG; (iv) Medium supplemented with 20% galactose; (v) Medium supplemented with 20% galactose and 0.5 mM MG; (vi) Medium supplemented with 20% galactose and 1 mM MG; (vii) Medium supplemented with 20% galactose and 2 mM MG; (viii) Medium supplemented with 20% galactose and 3 mM MG; (ix) Medium supplemented with 20% galactose and 4 mM MG. (C) The transformed cells were grown in liquid Ura- SD media supplemented with 20% galactose in presence of 1 mM and 4 mM MG. The growth of cells expressing *AtGLYI2* (shown in red color), *AtGLYI3* (shown in green color) and *AtGLYI6* (shown in purple color) was compared with that of cells expressing empty vector pYES2 (shown in blue color). The growth pattern was monitored spectrophotometrically for 30 h.

The *ΔGLO1* mutant BY4741 cells were transformed with pYES2-*AtGLYI2*, pYES2-*AtGLYI3*, pYES2-*AtGLYI6* and empty pYES2 vector. In order to confirm whether the transformed cells containing recombinant pYES2 vectors express the proteins or not, AtGLYI2, AtGLYI3 and AtGLYI6 protein specific antibodies were used to perform Western blotting. The *ΔGLO1* mutant cells transformed with pYES2-*AtGLYI2*, pYES2-*AtGLYI3* and pYES2-*AtGLYI6* gave a sharp band of approximately 27 kDa, 35 kDa and 43 kDa, respectively (Fig 5A). No band was observed in cells transformed with empty vector pYES2. The transformed cells containing recombinant and empty pYES2 vector were grown in solid Ura- synthetic defined media containing either 20% glucose or 20% galactose with different MG concentrations of 0.0, 0.5, 1.0, 2.0, 3.0 and 4.0 mM (Fig 5B). In the absence of MG, growth was seen in all transformed cells on the medium containing either glucose or galactose. The *ΔGLO1* mutant cells transformed with empty vector pYES2 were unable to grow in presence of MG, whereas cells containing pYES2 construct of *AtGLYI3* and *AtGLYI6* showed growth up to 1 mM MG. However, the *ΔGLO1* mutant cells containing recombinant pYES2-*AtGLYI2* were observed to grow till a higher MG concentration, i.e. 4 mM.

To further validate these results, all the transformed cells were grown in Ura- SD liquid media with 1 mM and 4 mM concentrations of MG. In the presence of 1mM and 4 mM MG, the *ΔGLO1* yeast cells with empty pYES2 vector could not grow, whereas the cells transformed with *AtGLYI2*, *AtGLYI3* and *AtGLYI6* showed different growth rates with different concentrations of MG (Fig 5C). The cells with *AtGLYI6* could just survive in 1 mM MG and the growth of cells expressing *AtGLYI3* was better as compared to the cells expressing *AtGLYI6*. Whereas, in presence of 4 mM MG, the growth of *ΔGLO1* cells with empty vector control or the cells expressing *AtGLYI3* and *AtGLYI6* was inhibited (Fig 5C). In contrast, cells expressing *AtGLYI2* were most tolerant even at higher concentration of MG. The above data indicates that all the three *AtGLYI* genes were able to functionally complement the yeast mutant and expression of *AtGLYI2* gene confers tolerance to yeast cells at high MG concentration.

## Discussion

Glyoxalase system is ubiquitously present in both prokaryotes as well as eukaryotes. GLYI is the first enzyme of Glyoxalase pathway and is broadly categorized into two metal activation classes i.e. Zn^2+^ dependent and non- Zn^2+^ dependent. The majority of GLYI enzymes that are characterized since the discovery of the glyoxalase system in 1913 are Zn^2+^ dependent holoforms [19,20]. The enzymes that belong to this metal activation class are believed to be predominantly eukaryotic in origin, for example, the GLYI enzymes from *H*. *sapiens* and *S*. *cerevisiae* [12,13]. A number of bacterial GLYI enzymes from *P*. *aeruginosa*, *N*. *meningitidis and Y*. *pestis* show a metal activation profile, where Zn^2+^ is non-activating and maximal enzyme activity was conferred by Ni^2+^ ion [13]. Further studies revealed that *P*. *aeruginosa* contained multiple GLYI encoding genes from both metal activation classes. This was the first report of a eubacterial species with several GLYI encoding genes, and also of an organism possessing GLYI enzymes from both metal activation classes [14]. Recently, a unique Ni^2+^ dependent and MG inducible *GLYI* was reported in rice [15]. These two distinct classes of GLYI enzymes have now been identified in *A*. *thaliana*. This co-existence of both the metal activation classes of GLYI enzyme in *A*. *thaliana* provides a unique opportunity for comparative study of both the enzyme classes for the first time in a higher eukaryote. In the present study carried out in *Arabidopsis thaliana*, we found three active GLYI enzymes (AtGLYI2, AtGLYI3 and AtGLYI6) belonging to two different metal activation classes. Protein sequence alignment of these three AtGLYI enzymes with the GLYI enzymes of other species showed presence of some extra amino acid sequences in AtGLYI2 (regions A, B and C in Fig 1) which is characteristic of Zn^2+^ dependent GLYI [14]. Absence of these extra amino acids from AtGLYI3 and AtGLYI6 indicates them to be non-Zn^2+^ dependent enzymes. In order to investigate about the metal dependency of these enzymes, we cloned, expressed and purified these proteins. AtGLYI2 was firstly demetalled and reconstituted with various metal ions. The reconstituted AtGLYI2 enzyme showed the maximal activity with MnCl_2_ followed by CoCl_2_, NiCl_2_ and CdCl_2_. This behavior is similar to that observed in GloA3 of *P*. *aeruginosa* [14] and is characteristic of Zn^2+^ dependent GLYI enzymes. Thus, this suggests that AtGLYI2 is a Zn^2+^ dependent GLYI. AtGLYI3 and AtGLYI6 are Ni^2+^ dependent enzymes as they showed maximal activity in the presence of Ni^2+^. These proteins had N-terminal Histidine tag incorporated in them and Histidine tag has high affinity for Ni^2+^, therefore Histidine tag of these two proteins was removed. GLYI activity of both proteins with or without Histidine tag was found similar to each other (Fig 2). This gave a clear indication that the observed GLYI activity of AtGLYI3 and AtGLYI6 was Ni^2+^ dependent and His tag independent. The metal activation profile of these enzymes clearly indicates the coexistence of three active different metal activated GLYI enzymes in Arabidopsis.

The kinetic profile of these enzymes reveals AtGLYI2 to be the most active GLYI. It is 250 times more active than AtGLYI3 and 670 times more active than AtGLYI6 (Table 1). Also the specific activity of AtGLYI2 is extremely high in comparison to the specific activity of AtGLYI3, AtGLYI6 and GLYI enzymes from other plant species such as Rice (*O*. *sativa)*, Onion (*A*. *cepa)*, Radish (*R*. *sativus)*, Carrot (*D*. *carota)* and Sweet potato *(I*. *batatas)* (Table 2). AtGLYI2 also has the highest activity among the other known Zn^2+^ dependent GLYI enzymes belonging to different species such as *P*. *aeruginosa*, *P*. *falciparum*, *S*. *cerevisiae* and *H*. *sapiens* (Table 3). A similar comparison of AtGLYI3 and AtGLYI6 with Ni^2+^ dependent GLYI enzymes from *O*. *sativa*, *P*. *aeruginosa*, *E*. *coli* and *C*. *acetobutylicum* reveals that AtGLYI3 and AtGLYI6 are the most active Ni^2+^ dependent enzymes (Table 4).

**Table 2:** Comparison of the specific activity of the GLYI proteins of Arabidopsis with GLYI proteins from other plant species.

Protein source	Specific activity (μmol/min/mg)	References
***A*. *thaliana* (AtGLYI2)**	**5157**	**This study**
***A*. *thaliana* (AtGLYI3)**	**20.7**	**This study**
***A*. *thaliana* (AtGLYI6)**	**7.68**	**This study**
*O*. *sativa* (OsGLYI-11.2)	120	[15]
*A*. *cepa* (GLYI)	4.54	[21]
*R*. *sativus* (GLYI)	2.19	[21]
*D*. *carota* (GLYI)	1.31	[21]
*I*. *batatas* (GLYI)	1.04	[21]

**Table 3:** A comparison of the kinetic parameters of AtGLYI2 (a Zn^2+^ dependent GLYI) with Zn^2+^ dependent GLYI from other species.

Protein Source	K_cat_ (s^-1^)	K_cat_/K_m_ (M^-1^/s^-1^)	Reference
***A*. *thaliana* (AtGLYI2)**	**13.76X10**^**4**^	**174.9X10**^**6**^	**This study**
*P*. *aeruginosa* (GloA3)	787	2.8X10^6^	[14]
*P*. *falciparum* (PfGlx I)	66.67	0.9X10^6^	[22]
*S*. *cerevisiae* (Glyoxalase-I)	1120	3.5X10^6^	[23]
*H*. *sapiens* (Glyoxalase I)	500	23X10^6^	[12]

**Table 4:** A comparison of the kinetic parameters of AtGLYI3 and AtGLYI6 (Ni^2+^ dependent GLYI) with Ni^2+^ dependent GLYI from other species.

Protein source	K_cat_ (s^-1^)	K_cat_/K_m_ (M^-1^/s^-1^)	Reference
***A*. *thaliana* (AtGLYI3)**	**728.8**	**16.08X10**^**6**^	**This study**
***A*. *thaliana* (AtGLYI6)**	**330**	**1.48X10**^**6**^	**This study**
*O*. *sativa* (OsGLYI-11.2)	70.96	0.71X10^6^	[15]
*P*. *aeruginosa* (GloA2)	247	12X10^6^	[14]
*E*.*coli* (GlxI)	338	12.4X10^6^	[24]
*C*. *acetobutylicum* (CLO GlxI)	1.38	0.26X10^6^	[25]

Heterologous expression of *AtGLYI* genes in *E*. *coli* conferred stress tolerance to the cells in response to different stress conditions (Fig 4). The growth of *AtGLYI* overexpressing cells was better than control. Among the three genes, *AtGLYI2* overexpressing cells were much more tolerant than *AtGLYI3* and *AtGLYI6* in presence to salinity (NaCl), oxidative (H_2_O_2_), exogenous MG, osmotic (mannitol) and heat stress. Thus, the overexpression of these GLYI genes provides multistress tolerance. AtGLYI2 has been found to provide considerable tolerance to stress conditions, as the cells overexpressing *AtGLYI2* were able to grow better in presence of 1 mM MG than the cells overexpressing *AtGLYI3*, *AtGLYI6* and empty vector control. The observed stress tolerance capacity of *E*. *coli* cells overexpressing *AtGLYI* genes correlates well with the kinetic parameters where AtGLYI2 was found to have much higher specific activity than AtGLYI3 and AtGLYI6.

All three *AtGLYI* genes also effectively complemented the growth defect of yeast *ΔGLO1* mutant cells in presence of exogenous MG. On solid Ura- media, cells transformed with *AtGLYI3* and *AtGLYI6* genes grew upto 1 mM MG concentration whereas cells with *AtGLYI2* exhibited tolerance even at 4 mM MG concentration. In liquid Ura- SD media supplemented with galactose, similar results were obtained. From the growth curve (Fig 5C), it can be concluded that AtGLYI3 and AtGLYI6 confer tolerance to exogenous MG stress in yeast cells.

However, the cells expressing *AtGLYI2* grew even at the higher concentration of MG (4 mM), a concentration much higher than the one considered lethal for yeast *GLO1* mutant cells (1 mM). In the report on Ni^+2^ dependent GLYI from rice, the OsGLYI-11.2 was found to complement *ΔGLO1* yeast mutants but they grew only upto 1 mM MG [15]. This clearly indicates that AtGLYI2 has quite high GLYI activity, which correlates very well with the kinetic data and the stress tolerance seen in *E*. *coli*. Also AtGLYI2 is a Zn^2+^ dependent GLYI enzyme, and contributes much more to the stress tolerance than the Ni^2+^ dependent GLYI enzymes, AtGLYI3 and AtGLYI6.

Till date, only *P*. *aeruginosa* has been known to contain multiple GLYI homologs belonging to both the metal activation classes. Now a eukaryote, *Arabidopsis thaliana* has been found to contain multiple active GLYI enzymes belonging to both Zn^2+^ and Ni^2+^ dependent metal activation classes. In plants some reports have described production of transgenic plants with improved tolerance to stress after overexpressing genes such as *GLYI* from *Brassica juncea* in tobacco [26] and in *Vigna mungo* [27], *GLYI* and *GLYII* in tobacco [10]. Moreover, similar tolerance to stress was observed when *GLYI* from wheat, sugar beet and rice plant was expressed in tobacco [15,28,29]. Thus, the glyoxalase pathway has a direct correlation with tolerance towards abiotic stresses. Overexpression of glyoxalase pathway enzymes can keep a check on the elevation of MG level and also helps in maintaining a higher “reduced to oxidized” glutathione ratio [30]. This may be the basis of improved abiotic stress tolerance of glyoxalase overexpressing transgenic plants reported previously and transformed bacterial cells in the present study. As AtGLYI2 has been found to be extremely active and also provides better stress tolerance to *E*. *coli*; it can be a probable candidate gene in terms of conferring multistress tolerance in plants. Although many studies have been undertaken to characterize the role of *GLYI* genes in plants, none of these have yet been successful in correlating the metal specificity with the role of GLYI in abiotic stress tolerance. We have also tried to establish a similar kind of relationship and have successfully correlated the higher activity of Zn^2+^ dependent AtGLYI2 with stress tolerance in bacterial cells. But, it is still unclear how Ni^2+^ dependent or Zn^2+^ dependent GLYI enzyme contributes towards stress tolerance in plants. Also the reason behind presence of multiple active GLYI enzymes in same organism and more contribution of Zn^2+^ dependent GLYI towards stress tolerance is not yet known. Since Arabidopsis has multiple active enzymes, it would be interesting to study their contribution in abiotic stress tolerance *in planta*. In future it would be interesting to study the overexpressing lines and mutant lines of these genes in Arabidopsis, in response to various abiotic stresses.

## Supporting Information

S1 Table A list of putative active GLYI members identified in Arabidopsis. Each of the eleven GLYI protein sequences were analyzed using BLASTP search tool for the presence of various domains required for GLYI activity. AtGLYI2, AtGLYI3 and AtGLYI6 were found to possess all four important domains.
(TIFF)

S2 Table A list of primers used in the study. (TIFF)
