# Correction: *Arabidopsis thaliana* Contains Both Ni^2+^ and Zn^2+^ Dependent Glyoxalase I Enzymes and Ectopic Expression of the Latter Contributes More towards Abiotic Stress Tolerance in *E*. *coli*

**DOI:** 10.1371/journal.pone.0162119

**Published:** 2016-08-29

**Authors:** The PLOS ONE Staff

The affiliation for the second author is incorrect. Rituraj Batth is not affiliated with #2 but with #1 Faculty of Life Sciences and Biotechnology, Plant Molecular Biology Laboratory, South Asian University, Akbar Bhawan, Chanakyapuri, New Delhi 110021, India. The publisher apologizes for the error.
