# Supplementary figures and images for: Arabidopsis thaliana Contains Both Ni2+ and Zn2+ Dependent Glyoxalase I Enzymes and Ectopic Expression of the Latter Contributes More towards Abiotic Stress Tolerance in E. coli
Source: PLoS One. 2016 Jul 14;11(7):e0159348. doi: 10.1371/journal.pone.0159348 (PMC4945007; doi:10.1371/journal.pone.0159348)

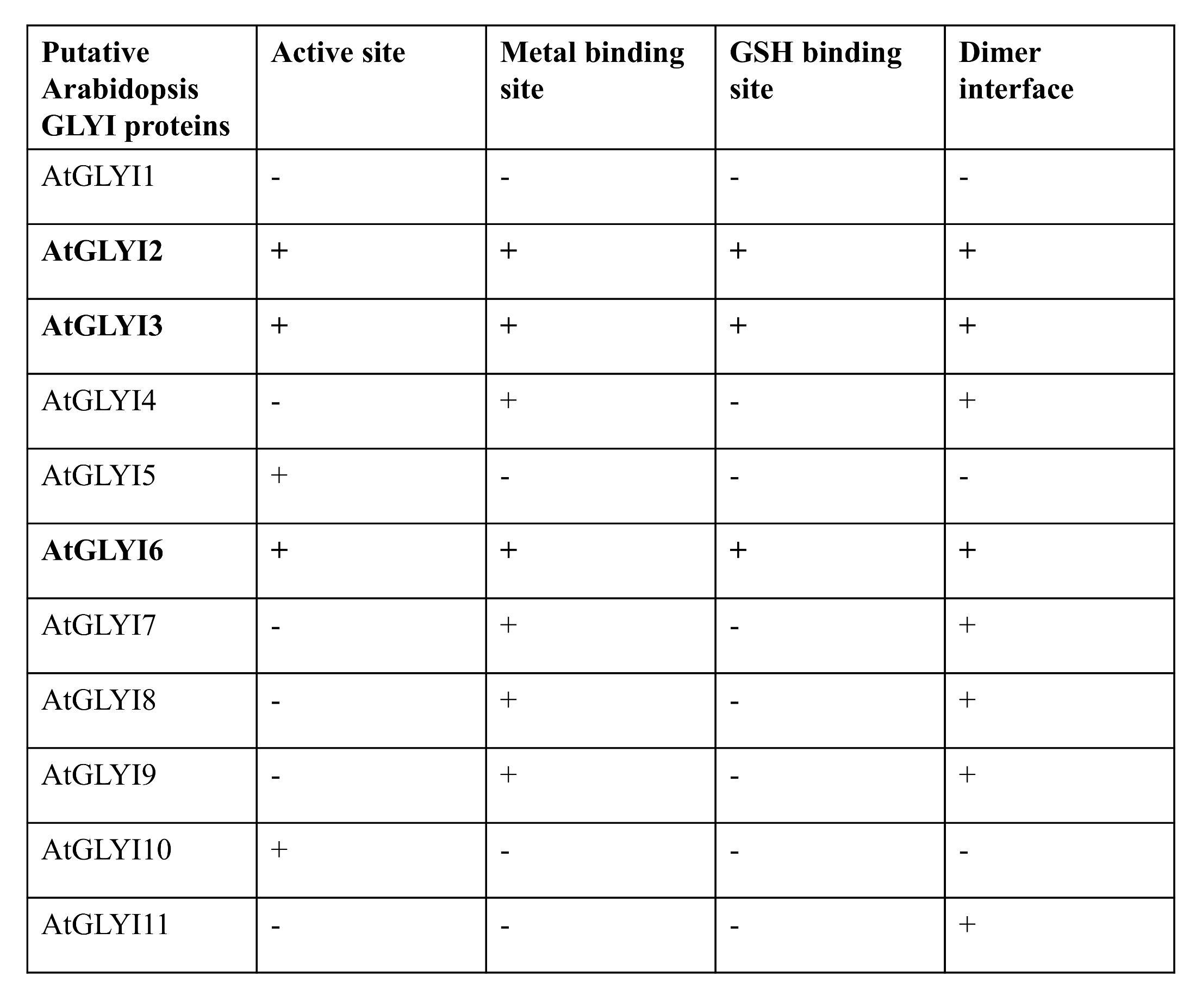

Supplement: S1 Table — Each of the eleven GLYI protein sequences were analyzed using BLASTP search tool for the presence of various domains required for GLYI activity. AtGLYI2, AtGLYI3 and AtGLYI6 were found to possess all four important domains. (TIFF) [file pone.0159348.s001.tiff]

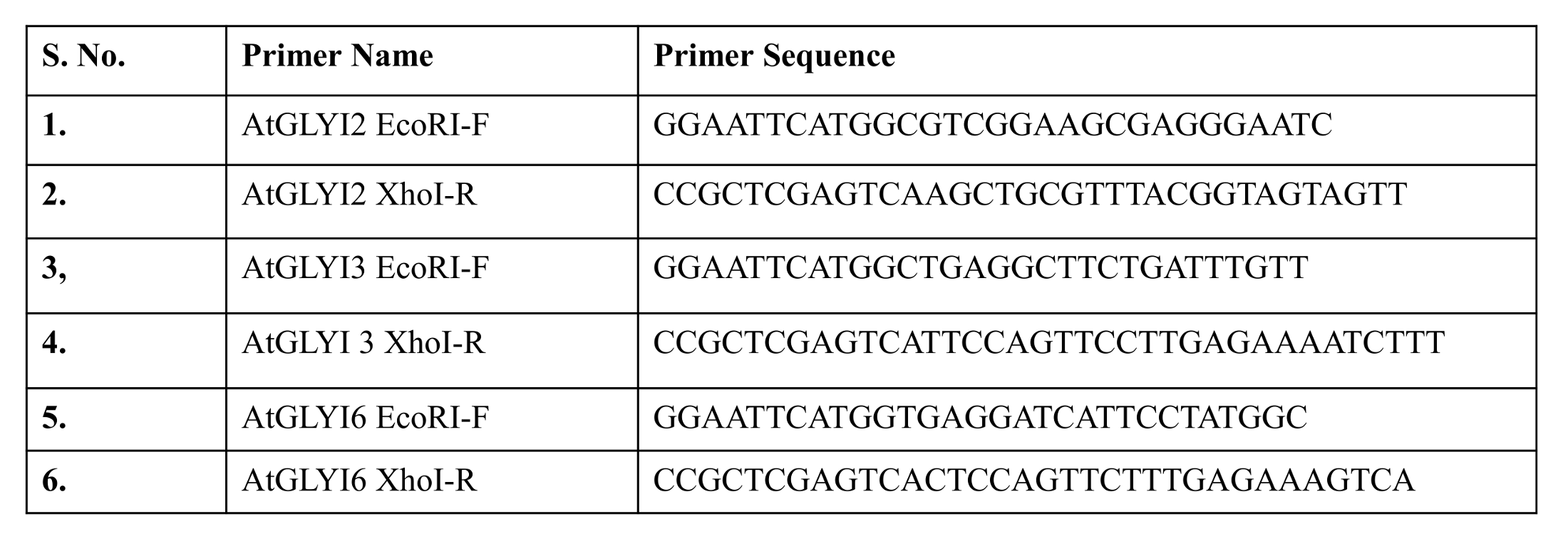

Supplement: S2 Table — (TIFF) [file pone.0159348.s002.tiff]
